# Factors associated with decision-making on prophylactic hysterectomy and attitudes towards gynecological surveillance among women with Lynch syndrome (LS): a descriptive study

**DOI:** 10.1007/s10689-020-00158-5

**Published:** 2020-01-29

**Authors:** Mari H. Kalamo, J. U. Mäenpää, T. T. Seppälä, J. P. Mecklin, H. Huhtala, K. Sorvettula, K. Pylvänäinen, S. Staff

**Affiliations:** 1 Department of Gynecology and Obstetrics and Cancer Center, Tampere University Hospital, Tampere, Finland; 2 Faculty of Medicine and Medical Technology, Tampere University, Tampere, Finland; 3 Department of Gastrointestinal Surgery, Helsinki University Hospital and University of Helsinki, Helsinki, Finland; 4 Department of Surgery, Central Finland Central Hospital, Jyväskylä, Finland; 5 Faculty of Sports and Health Sciences, University of Jyväskylä, Jyväskylä, Finland; 6 Department of Education and Science, Central Finland Health Care District, Jyväskylä, Finland

**Keywords:** Lynch syndrome, HNPCC, Surveillance, Prophylactic surgery

## Abstract

To prevent endometrial carcinoma in Lynch syndrome (LS), regular gynecological surveillance visits and prophylactic surgery are recommended. Previous data have shown that prophylactic hysterectomy is an effective means of cancer prevention, while the advantages and disadvantages of surveillance are somewhat unclear. We aimed to evaluate female LS carriers’ attitudes towards regular gynecological surveillance and factors influencing their decision-making on prophylactic surgery that have not been well documented. Pain experienced during endometrial biopsies was also evaluated. Postal questionnaires were sent to LS carriers undergoing regular gynecological surveillance. Questionnaires were sent to 112 women with LS, of whom 76 responded (68%). Forty-two (55%) had undergone prophylactic hysterectomy by the time of the study. The majority of responders (64/76; 84.2%) considered surveillance appointments beneficial. Pain level during endometrial biopsy was not associated with the decision to undergo prophylactic surgery. The level of satisfaction the women had with the information and advice provided during surveillance was significantly associated with the history of prophylactic hysterectomy (satisfaction rate of 73.2% versus 31.8% of nonoperated women, *p* = 0.003). The women who had undergone prophylactic surgery were older than the nonoperated women both at mutation testing (median of 42.3 years versus 31.6 years, *p* < 0.001) and at the time of the study (median of 56.9 years versus 46.0 years, respectively, *p* < 0.001). Women with LS pathogenic variants have positive experiences with gynecological surveillance visits, and their perception of the quality of the information and advice obtained plays an important role in their decision-making concerning prophylactic surgery.

## Introduction

Lynch syndrome (LS), previously called hereditary nonpolyposis colorectal cancer (HNPCC), is a cancer predisposition syndrome with a dominant inheritance caused by pathogenic (path_) germline variants in the DNA mismatch repair (MMR) genes *MLH1*, *MSH2*, *MSH6*, and *PMS2* [1]. In addition to the early occurrence of colorectal cancer (CRC), LS is also characterized by certain extracolonic cancers (ECCs), of which endometrial carcinoma (EC) is the most common [1]. Carriers of different *path_MMR* variants exhibit distinct patterns of cancer risk and survival. The cumulative incidence of EC for *path_MLH1*, *path_MSH2*, *path_MSH6* and *path_PMS2* is 42.7%, 56.7%, 46.2% and 26.4% at the age of 75 years, respectively [2].

ECs associated with *path_MMR* variants usually occur at younger ages than in the general population. The average age at EC diagnosis in women with LS in a recent retrospective series was reported to be 47–49 years (range 26–87) [1, 3]. The steepest increase in the cumulative incidence of EC was between 50 and 60 years of age in the Prospective Lynch Syndrome Database (PLSD) [2].

The clinical practice and guidelines for gynecological surveillance and prophylactic surgery for female LS variant carriers vary in different countries [4]. Common practice in countries performing surveillance in Europe, Australia, North America and South America is either annual on biannual gynecological examination [5]. Based on current published studies, there are no adequate data for evidence-based clinical decisions based on findings during surveillance [6]. In Finland, after predictive genetic testing was nationally introduced in 1995, annual gynecological examinations have become common clinical practice, including pelvic ultrasound examination and endometrial biopsy, starting at approximately 35 years of age [7]. Prophylactic surgery, or hysterectomy with or without bilateral salpingo-oophorectomy or salpingectomy, has usually been performed after the age of 40 years, when having children is complete, or at the age of menopause, depending on the mutation carrier’s preference [3, 4]. However, some pathogenic variant carriers disagree with the surgery recommendation and refuse to undergo prophylactic hysterectomy. The cancer-preventing effects of prophylactic surgery have been proven by clinical trials [6]. A few previous studies have evaluated the process of decision-making on prophylactic surgery, the effects of gynecological surveillance and prophylactic hysterectomy on the quality of life, and the pain associated with endometrial sampling of the mutation carriers [4, 8–10].

Since data on the attitudes of LS mutation carriers towards prophylactic surgery and gynecological surveillance are limited and even absent in Finland, we wanted to evaluate the decision-making process, satisfaction with surveillance, and pain associated with endometrial biopsies in this questionnaire-based study.

## Materials and methods

### Study subjects

The present retrospective cohort study was performed at Tampere University Hospital (TAUH), Tampere, Finland. Informed consent was obtained from all study participants, and the study protocol was approved by the TAUH Ethical Committee (decision code ETL R10079, dated 4.1.2011).

The study cohort included Finnish women with inherited pathogenic MMR gene variants identified from the nationwide Finnish LS Registry (LSRFi), [11] which has been described in more detail previously [12, 13, 14, 15]. Briefly, the LSRFi includes 300 families and approximately 1400 verified germline MMR variant carriers (http://www.hnpcc.fi/). Healthy women belonging to a Finnish LS family receive counseling from clinical geneticists and gynecologists. After counseling, the decision to undergo mutation testing and its timing are based on the woman’s individual choice. Regular follow-up of the mutation-positive women starts after the mutation testing. Prophylactic hysterectomy is generally recommended for all female mutation carriers after 35–40 years of age, when the mutation carrier is no longer wishing for a pregnancy. Surgery is recommended by the age of menopause at the latest. If prophylactic surgery has not been performed by the age of 40, annual follow-up visits are recommended. The removal of the ovaries is discussed with mutation carriers and is usually performed if the woman is peri- or postmenopausal or if she, after receiving information, decides to have them removed before menopause. Finally, salpingo-oophorectomy is recommended at the time of menopause at the latest.

One hundred and twelve female LS carriers at least 30 years of age, with no history of endometrial or ovarian cancer and having previously consented regarding registry inquiries, were identified from the LSRFi. The study cohort is described in Table 1. A postal questionnaire was sent to these 112 women and was re-sent to those who did not return questionnaires within 6 months of the first mailing.

**Table 1:** Characteristics of the LS cohort (112 Finnish females with a *path_MMR* variant)

	Whole LS cohort (N = 112)	Study population (responders) (N = 76)
Age		
Median (range)	49 (30–89)	52 (30–82)
Age at mutation testing		
Median	38 (20–72)	36 (22–65)
Distribution of MMR genes		
* MLH1*	72 (64%)	47 (62%)
* MSH2*	32 (29%)	22 (29%)
* MSH6*	8 (7%)	7 (9%)
History of other cancer (Y/N)		
Y	42 (38%)	24 (33%)
N	70 (62%)	49 (67%)
Prophylactic surgery performed		
Y	63 (56%)	42 (68%)
N	49 (44%)	24 (32%)

*LS* Lynch syndrome, *path_MMR* pathogenic variant of DNA mismatch repair gene

### Questionnaires

Study participants completed a retrospective questionnaire collecting data on their history of other types of cancer, family history, parity, and age at mutation testing and prophylactic gynecological surgery, if performed. Data on the subjects’ attitudes towards gynecological surveillance and prophylactic surgery and their experiences with these procedures were also collected. Pain associated with endometrial biopsies was evaluated with a numeric rating scale (NRS; 0–10, 0 = no pain and 10 = worst imaginable pain). Subjects who stated they could not recall or evaluate the pain level did not answer this question. A detailed description of the questionnaire content is presented in Table 2.

**Table 2:** Details of the questionnaire used

Feature	Further information	Measurement/response
Time of predictive testing		Date/year
Age at predictive testing		Number
Relationship status before testing	In a relationship?	Y/N
Relationship status on study	In a relationship?	Y/N
Prophylactic surgery performed		Y/N
Has attended follow-up appointments		Y/N
Considers follow-up beneficial	If “yes” to previous	Y/N
Parity	Number of deliveries	Number
Experienced pain in endometrial biopsy	NRS 0–10	Number
Satisfied with the advice provided by the professionals	In general	Y/N
Enough information provided on possible adverse effects of prophylactic surgery	Gynecological prolapses	Y/N
	Urinating complaints	Y/N
	G-I tract complaints	Y/N
Has felt pressure for prophylactic surgery		Y/N
Satisfied with decision to have surgery	If performed	Y/N
Planning to have prophylactic surgery	If not performed	Y/N
Cancer other than gynecological cancer in family	Personal history or family member	Y/N
	Which cancer	Description
Family member died of gynecological cancer		Y/N
Experience of personal state of health	Poor/intermediate/good	0/1/2
Poor tolerance of insecurity	Own experience	Y/N
Strong fear of cancer		Y/N
Strong fear of surgery/operations		Y/N
Experience of surgery as responsibility		Y/N

### Statistical analysis

IBM SPSS statistics software, version 22 (IBM SPSS, Inc., Armonk, NY, USA), was used for the statistical analyses. The association of categorized variables with prophylactic surgery decisions was performed using the chi-squared test. Two-tailed *P* < 0.05 values were considered to indicate statistically significant differences. The association of continuous variables (e.g., NRS and number of deliveries) with the history of prophylactic surgery was carried out using the t test or a nonparametric test when appropriate. NRS scores and the number of deliveries were also categorized (NRS 0 to 5 versus 6 or more and parity of 0 versus 1 or more deliveries) in the statistical analyses. When assessing factors possibly influencing prophylactic surgery decision-making, patients who had a hysterectomy for nonprophylactic reasons were excluded from the analyses.

## Results

Seventy-six women returned the questionnaire, resulting in a 68% response rate. The distribution of the affected genes was as follows: 62% *MLH1*, 29% *MSH2* and 9% *MSH6* mutations. A prophylactic hysterectomy was performed on 42 subjects of this population (55%) at the median age of 42.0 years (range 32.0–67.0). Twenty-four subjects had not had a hysterectomy at the time of the survey, and 10 subjects had a nonprophylactic hysterectomy performed for benign medical reasons, such as uterine myomas, menorrhagia without endometrial hyperplasia, and pelvic floor prolapses, for which they were excluded from the analyses concerning prophylactic hysterectomy. The characteristics of the study cohort (both responders and nonresponders) are summarized in Table 1.

Among subjects not having had a hysterectomy performed at the time of the study, eight (33.3%) reported they were not planning to have a prophylactic hysterectomy at all, and 16 (66.7%) reported not having decided yet about the surgery or did not respond.

The median age at mutation testing among subjects with a prophylactic hysterectomy performed was 42.3 years (range 25–65), compared to 31.6 years (range 22–48) for subjects with no hysterectomy performed (*p* < 0.001). At the time of the study, the median age of subjects with a prophylactic hysterectomy performed was 56.9 years (range 43–72), compared to 43.2 years (range 30–76) for subjects with no hysterectomy performed (p < 0.001).

The median time interval between mutation testing and the study survey was 11 years (range 6–29 years) among the study subjects still in surveillance (not having undergone prophylactic surgery). The median duration of surveillance (median time interval between mutation testing and prophylactic surgery) was 6 years (0–14 years), and the median time interval between surgery and the study questionnaire was 9 years (1–38 years) among the prophylactically operated subjects.

Sixty-eight (89.5%) of the responders reported attending regular surveillance appointments that were provided. Six subjects reported not having been offered appointments at all, and two subjects did not respond to this question. Sixty-four (84.2%) of the subjects considered appointments to be beneficial, 10 subjects did not respond to this question and only two patients considered appointments unbeneficial.

Pain associated with endometrial sampling measured by NRS, overall satisfaction with the given information and all the background factors possibly having an influence on women’s attitudes and decisions on prophylactic surgery obtained from the questionnaires are summarized in Table 3. Fifty-four subjects evaluated pain associated with endometrial biopsy, while 22 (29%) of the subjects did not respond to this question. The median NRS among Women with LS was 3.5. Most women (72.2%) reported mild or intermediate pain associated with endometrial biopsy measured by NRS (NRS 0–5), and strong pain (NRS 6–10) was reported by 27.8% of women. Approximately 40% of participants reported pain to be very mild or there was no pain at all (NRS 0 to 2). Pain levels during endometrial biopsy did not influence the rate of prophylactic surgeries when analyzed either as a continuous variable or when categorized. Regardless of the history of prophylactic hysterectomy, a majority of women (43/76; 59.7%) reported satisfaction with the information and advice regarding LS in general and prophylactic surgery provided by gynecologists. Only four subjects did not answer this question. The self-reported satisfaction with general LS-associated information and advice by experts was dependent on the history of prophylactic hysterectomy: 73.2% of the operated patients were satisfied versus only 31.8% of the nonoperated patients (p = 0.003; Table 3). The compliance rate with gynecological surveillance was similar among operated and nonoperated women (88.1% versus 87.5%, respectively, p = 1.00; Table 3).

**Table 3:** Background characteristics and factors collected from questionnaires obtained from prophylactically operated vs. nonoperated mutation carriers

Reported variables	Study population (N = 76)^a^	Prophylactic hysterectomy performed (N = 42)^b^	Nonoperated (N = 24)	p value^c^
	n (%)	n (%)	n(%)	
1. Parity: 1 or more deliveries	66 (86.8)	37 (88.1)	21 (87.5)	1.000
2. Own health considered intermediate or good	50 (65.8)	24 (58.5)	18 (75.0)	0.282
3. In a relationship at mutation diagnosis	67 (88.2)	40 (95.2)	19 (79.2)	0.089
4. Attended gynecological appointments regularly	68 (89.5)	37 (88.1)	21 (87.5)	1.000
5. Cancer other than gynecological cancer in family	73 (96.0)	39 (92.9)	24 (100.0)	0.295
6. Family member died of gynecological cancer	17 (22.3)	12 (29.3)	4 (16.7)	0.373
7. Poor tolerance of feeling of insecurity	13 (17.1)	5 (11.9)	5 (20.8)	0.477
8. Strong fear of cancer	32 (42.1)	19 (45.2)	10 (41.7)	0.803
9. Strong fear of surgery/operations	12 (15.7)	6 (14.3)	4 (16.7)	1.000
10. Experience of surgery as responsibility	16 (21.0)	12 (29.3)	3 (12.5)	0.142
11. Feels/has felt pressure for surgery	20 (26.3)	14 (35.9)	5 (22.7)	0.391
12. Satisfied with information and advice in general	43 (56.6)	30 (73.2)	7 (31.8)	0.003
13. Satisfied with information on possible postoperative disadvantages				
a. Urinary complaints	18 (23.6)	11 (28.9)	2 (9.1)	0.106
b. Chronic pelvic pain	20 (26.3)	12 (31.6)	3 (13.6)	0.215
c. GI-tract complaints	19 (25.0)	12 (31.6)	2 (9.1)	0.061
d. Pelvic prolapses	19 (25.0)	12 (31.6)	2 (9.1)	0.061
14. Endometrial biopsy pain (NRS score)	(N = 54)^d^	(N = 28)^d^	(N = 19)^d^	
a. 0–5	39 (72.2)	21 (75.0)	11 (57.9)	
b. 6–10	15 (27.8)	7 (25.0)	8 (42.1)	0.339
15. Median age, years	Year (range)	Year (range)	Year (range)	
a. At mutation testing	35.2 (22–65)	42.3 (25–65)	31.6 (22–48)	< 0.001
b. At survey	48.8 (30–76)	56.9 (43–72)	43.2 (30–76)	< 0.001

^a^Defined by study questionnaire responders

^b^10 subjects with nonprophylactic hysterectomy excluded from comparison

^c^Comparison between nonoperated and prophylactically operated subjects

^d^Total of 54 subjects answered this question (22 subjects did not respond)

In addition, there was a trend for women who chose prophylactic hysterectomy to have received more information on certain postoperative complications than women who had not chosen surgery yet (p = 0.061 for information on GI-tract postoperative complications and pelvic prolapses; Table 3).

## Discussion

In this study, LS pathogenic variant carriers’ attitudes towards gynecological surveillance and satisfaction with the advice and information provided by experts were significantly associated with having had prophylactic surgery. To our knowledge, this finding highlights the importance of general information in this context and emphasizes the role of attending medical staff. On the other hand, compliance with surveillance was similar between prophylactically operated and nonoperated women, suggesting that the quality of information may play a significant role in decision-making.

Parity and experienced pain during endometrial sampling did not correlate with the decision to undergo prophylactic surgery. A previous study indicated that parity influences decision-making, but the data were derived from a very small study population of ten women with LS [8]. Severe pain experienced during endometrial sampling has been previously shown to be the main reason to quit screening, thus possibly lowering the threshold for surgery [10]. Different populations may explain differences in the results concerning the experience of pain during endometrial sampling. Since ultrasound examination is not sufficient as a single surveillance method in terms of EC prevention, [16] it is a relief that pain associated with endometrial sampling was not a significant factor for decision-making, at least in our study population. The association of older age at mutation testing and at survey was expected since all recommendations for the initiation of surveillance and the timing of prophylactic surgery are age-dependent.

The majority of subjects considered gynecological surveillance to be beneficial in general. There have been some previous qualitative studies on the topic showing experienced benefit [8, 10]. We show that some of the study subjects reported being either inadequately or not at all informed about the risks and possible long-term side effects of prophylactic surgery. Earlier qualitative studies evaluating surgery decisions have reported similar results: mutation carriers are mainly satisfied with prophylactic surgery decisions, but nonoperated women are not completely satisfied with the information they receive [6, 8]. One possible explanation for this is that more detailed surgery-related information is provided only when the decision to undergo surgery has been made. From this retrospective analysis, it is not possible to draw straightforward conclusions, but it is probable that women with LS may warrant more detailed and structured information on surgery during surveillance.

Some of our study subjects were not satisfied with the surveillance protocol. A few LS carriers reported not being informed at all about gynecological surveillance appointments. This probably influenced their decision-making on prophylactic surgery and may have led them to refuse it, thus keeping them susceptible to EC. This finding emphasizes the importance of structured national guidelines for the management of LS.

The strengths of our study include a well-defined population of women with LS who were all verified as germline pathogenic variant carriers and were not just women who had a strong family history of EC or CRC. The study cohort identified from the LSRFi included 112 women, and the response rate was quite high (68%), which is in line with previous questionnaire-based studies among subjects with a hereditary cancer predisposition [14, 15].

There are some limitations to our study. The setting is retrospective, and a questionnaire survey is subject to the risk of misremembering background factors. This misremembering may therefore cause recall bias. However, we consider that a questionnaire-based survey is also a valuable method to collect the points of view and experiences of women with LS. Prophylactically operated subjects were expectedly significantly older at the time of mutation diagnosis and at study than nonoperated women, which can also cause some bias. A comparison of responders to nonresponders did not reveal any major concerns other than the slightly more frequent rate of prophylactic hysterectomy among the responders, which may cause potential bias and must be taken into account when interpreting the present results. Some of the study subjects had a hysterectomy for nonprophylactic reasons, and they had to be excluded from the analyses when estimating the factors influencing the decision-making about prophylactic surgery.

In conclusion, we show here new descriptive data on the attitudes towards surveillance and factors associated with the history of prophylactic surgery in a Finnish cohort of women with LS. Based on our results, surveillance is well accepted. Considering the results of our study, we suggest that the mutation carriers should be systematically informed about surveillance and its aims and about prophylactic surgery. We suggest that information should be offered regardless of the timing of the prophylactic surgery.
